# Tofacitinib Decreases Autophagy of Fibroblast-Like Synoviocytes From Rheumatoid Arthritis Patients

**DOI:** 10.3389/fphar.2022.852802

**Published:** 2022-03-03

**Authors:** M. Vomero, M. Caliste, C. Barbati, M. Speziali, A. I. Celia, F. Ucci, C. Ciancarella, E. Putro, T. Colasanti, G. Buoncuore, E. Corsiero, M. Bombardieri, F. R. Spinelli, F. Ceccarelli, F. Conti, C. Alessandri

**Affiliations:** ^1^ Arthritis Center, Dipartimento di Scienze Cliniche Internistiche, Anestesiologiche e Cardiovascolari, Sapienza University of Rome, Rome, Italy; ^2^ Rheumatology, Immunology and Clinical Medicine Unit, Università Campus Bio-Medico di Roma, Rome, Italy; ^3^ Centre for Experimental Medicine and Rheumatology, William Harvey Research Institute, Queen Mary University of London, London, United Kingdom

**Keywords:** Rheumatoid arthritis, tofacitinib, janus tyrosine kinases, autophagy, apoptosis

## Abstract

The pathway of Janus tyrosine kinases (JAKs) has a central role in the pathogenesis of Rheumatoid Arthritis (RA) by regulating multiple immune functions and cytokine production. The JAK inhibitor tofacitinib is effective in RA patients not responding to methotrexate or TNF-inhibitors. Since hyperactive autophagy has been associated with impaired apoptosis of RA fibroblast-like synoviocytes (FLS), we aimed to investigate the role of tofacitinib in modulating autophagy and apoptosis in these cells. FLS isolated from RA biopsies were cultured with tofacitinib in presence of autophagy inducer rapamycin and in serum deprivation condition. Levels of autophagy, apoptosis, and citrullinated proteins were analyzed by western blot, flow cytometry, immunocytofluorescence, and Real-Time PCR. Rapamycin induced an increase in RA-FLS autophagy while the levels of autophagy marker LC3-II were reduced after *in vitro* treatment with tofacitinib. The analysis of autophagic flux by specific fluorescence dye confirmed the reduction of autophagy in RA FLS. The treatment with tofacitinib did not influence apoptosis of RA FLS. Modulation of the autophagic process by tofacitinib did not significantly change citrullination. The results of this study demonstrate that tofacitinib is able to modulate autophagy of FLS contributing to its effectiveness in RA patients.

## Introduction

Rheumatoid arthritis (RA) is an inflammatory disease characterized by an autoimmune response against self-antigens promoted by adaptive and innate immune cells. RA fibroblast-like synoviocytes (RA-FLS) are subjected to complex molecular changes that lead to an aggressive and invasive phenotype characterized by the release of inflammatory cytokines, chemokines and matrix-degrading enzymes (McInnes and Schett, 2020).

Autophagy is a metabolic process involved in the degradation of intracellular components via lysosomal machinery. Although several autophagy types are described in mammalian cells, macro-autophagy, hereafter referred to as autophagy, is the most studied (Nakatogawa et al., 2009). Autophagy is stimulated by starvation and it is characterized by proteins and organelles engulfment in double-membrane vesicles called autophagosomes. Later, the autophagosome fusion with lysosome ensures cargo degradation by lysosomal hydrolases and recycling cytoplasmic material to generate energy. Autophagy-related proteins provide the ongoing process and microtubule-associated protein light chain 3 (LC3) is widely considered to be the most crucial autophagy marker (Klionsky et al., 2021). A dysregulated autophagy has been implicated in the pathogenesis of several inflammatory diseases, including RA (Vomero et al., 2018). In RA-FLS, autophagy is persistently activated leading to chronic inflammation and joint damage. Moreover, RA-FLS showed apoptosis resistance, and increasing evidence supports the hypothesis that defective apoptosis in RA-FLS could result from hyperactivation of autophagy (Shin et al., 2010; Connor at al., 2012). The pathway of Janus tyrosine kinases (JAKs) regulates multiple immune functions and cytokine production involved in the pathogenesis of RA. Tofacitinib is the first generation JAK inhibitor approved by the FDA and EMA for the treatment of RA. The “cross-talk” between JAK/STAT and the stress-activated PI3K/AKT/mTOR axis, involved in autophagy regulation, seem to be dysregulated in RA (Malemud, 2013). Ireland et al. showed that the addition of an inhibitor of Phosphatidylinositide-3 kinase (PI3K), a downstream step of the JAK pathway, can inhibit the autophagy (Ireland and Unanue, 2011). Nevertheless, there are no other studies on the effect of JAK inhibition on autophagy.

Considering the central role of JAK pathway in the control of RA inflammatory response, we investigated the effects of tofacitinib on autophagy and apoptosis of RA-FLS.

## Methods

### RA-FLS Isolation

Synovial tissue was drawn during a total knee/hip knee replacement at Queen Mary University of London. After informed consent, samples from five RA patients (patients mean age was 73.25 years and 50% of patients were female) and from five osteoarthritis (OA) patients, used as control group, were obtained. FLS were purified from synovial tissue after digestion with dispase at 37°C for 60 min as previously described (Vomero et al., 2019). FLS were used between passages four and eight to avoid lymphocytes and macrophages contamination.

### Culture Condition and Treatments

For *in vitro* experiments, FLS were grow in a Dulbecco’s modification of Eagle medium (DMEM) supplemented with 10% Fetal Bovine serum (FBS, EuroClone) heat inactivated at 50°C for 30′, 50 IU/ml penicillin/streptomycin, 2 mM glutamine, and 10 mM HEPES, in a controlled atmosphere incubator at 37°C. Cells were subjected to different treatments.

- tofacitinib (provided by Pfizer) resuspended in DMSO and then diluted in cell medium at a concentration of 1 μM;
- rapamycin resuspended in DMSO and then diluted in cell medium at a concentration of 200 nM (Sigma-Aldrich);
- nutrients deprivation (starvation) obtained by culturing cells at a lower serum concentration (2% FBS).

After 24 h of culture, FLS were collected for analysis of autophagy and apoptosis as described below. Preliminary experiments on FLS isolated from patients affected by OA were performed (Supplementary Figure S1).

The study was approved by the Ethics Committee of Sapienza University of Rome (protocol number 707/17).

### Analysis of Autophagy

Autophagy levels in FLS were analyzed by evaluating the most common autophagy marker LC3-II by Western blot.

In detail, cells were lysed in RIPA buffer (100 mM Tris-HCL pH 8, 150 mM NaCl, 1% Triton X-100, 1 mM MgCl, 25 mM NaVO4 and complete protease-inhibitor mixture). After SDS-PAGE, western blot was performed using a rabbit anti-human LC3IIB Ab (Cell Signaling) and a rabbit anti-human p62 Ab (Sigma-Aldrich). Peroxidase-conjugated goat anti-rabbit IgG (Bio-Rad) were used as a secondary Ab and the reaction was developed using the SuperSignal West Pico Chemiluminescent Substrate (Pierce). To ensure the presence of equal amounts of protein a rabbit anti-human β-actin Ab (Sigma-Aldrich) was used. Quantification of protein expression was performed by densitometry analysis.

Autophagy flux was also measured by flow cytometry by using the Cyto-ID™ Autophagy Detection Kit (Enzo Life Science) as previously described (Colafrancesco et al., 2020).

Briefly, FLS were pelleted and then resuspended in DMEM without phenol red, supplemented with 10% FBS, and diluted Cyto-ID solution. After resuspension, the samples were incubated 30 min at 37°C in the dark. After centrifugation, samples were resuspended in assay buffer and transferred into FACS tubes. FACS Calibur (Becton and Dickinson) was employed for all measurement, 50,000 events/sample were run, and data were analysed using the Cell Quest Pro software (BD BioSciences).

For autophagy evaluation by immunocytofluorescence, FLS were cultured on the slides for 24 h to let them attach and, after treatments, incubated with the anti-human LC3B antibody overnight at 4°C (Cell Signaling), with the secondary antibody 30 min (Alexa 555 Goat anti-Rabbit IgG) and finally with DAPI (Sigma-Aldrich) for 15 min. Fluorescence was analyzed by a fluorescence microscope (Olympus BX52). Image acquisition and processing were conducted by IAS 2000 software. MFI has been measured in four different areas for each condition and normalized on the number of cells, as an expression of the number of autophagy vesicles.

### Analysis of Apoptosis by Flow Cytometry and Quantitative Real-Time PCR

The quantitative evaluation of apoptosis was performed after *in vitro* treatment with tofacitinib using Annexin V (AV) and Propidium iodide (PI) apoptosis detection kit according to the manufacturer’s protocol (Marine Biological Laboratory). The reported data were referred to AV-positive apoptotic cells.

Bad and Bax gene expression was used to quantify apoptosis in qRT-PCR. The RNA was extracted by FLS using the Arcturus Picopure RNA Isolation kit and reverse-transcribed in cDNA by SuperScript IV First-Strand cDNA Synthesis Reaction kit (Thermo Fisher Scientific). For the Real-Time PCR, the cDNA was mixed with dNTPs, Mg++, Taqman probes, and Taq polymerase, and finally amplified in 40 cycles (QuantStudio5 Real-Time PCR System). Probes used are: Bax (Thermo fisher Hs00180269_m1), Bad (Thermo fisher Hs00188930_m1), GAPDH (Thermo fisher Hs02786624_g1). The gene expression has been analyzed in a relative quantification with the ΔCt, as the difference between the housekeeping GAPDH cycle threshold (Ct) and the ones of our genes of interest.

### Western Blot Analysis of Citrullinated Proteins

FLS treated with tofacitinib/rapamycin alone or in combination, were lysed in lysis buffer and equal amounts of whole-extract proteins were subjected to 10% SDS-PAGE. The proteins were blotted onto PVDF membranes (Bio-Rad) and incubated with specific rabbit polyclonal anti-vimentin citrulline Ab (Millipore). Peroxidase-conjugated goat anti-rabbit IgG (Bio-Rad) were used as a secondary Ab and the reaction was developed using the SuperSignal West Pico Chemiluminescent Substrate (Pierce). Quantification of protein expression was performed by densitometry analysis.

### Statistical Analysis

Statistical analysis was performed using GraphPad Prism Version 6 (GraphPad Software, San Diego, CA, United States). Data are expressed as mean ± standard deviation (SD). Data were evaluated using normality test and analyzed using the Mann-Whitney test. *p* < 0.05 was considered to be statistically significant.

## Results

### *In Vitro* Tofacitinib Inhibited Autophagy, but not Apoptosis, of RA-FLS

**FIGURE 1:**
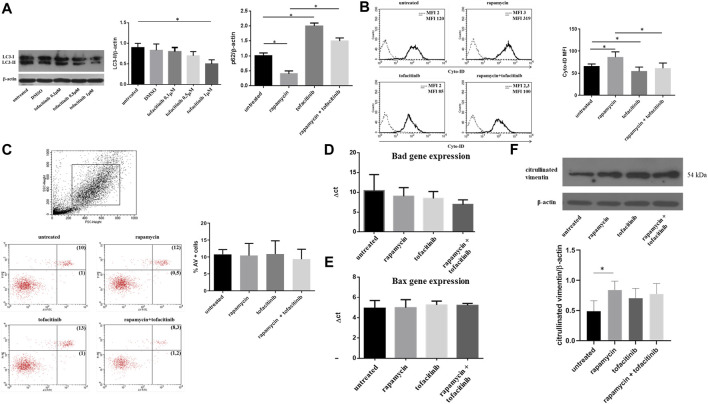
Autophagy, apoptosis and citrullinated proteins levels after *in vitro* treatment with tofacitinib in FLS isolated from RA patients. **(A)**. Western blot analysis of LC3-II and p62 levels of RA-FLS treated with tofacitinib (1uM) alone and in combination with rapamycin (200 nM). Blots shown are representative of five independent experiments performed using FLS samples from five different RA patients. Densitometry analysis of LC3-II and p62 levels relative to β-actin is also reported. Values are expressed as means ± sd. *p < 0.05 **(B)**. Analysis of autophagosomes number in FLS treated with tofacitinib as previously described (*n* = 5). Values of mean fluorescence intensity (MFI) obtained by flow cytometry are reported. *p < 0.05 **(C)**. Statistical analysis of apoptosis of FLS isolated from patients with RA (*n* = 5) after *in vitro* treatment with tofacitinib. Results are expressed as AV-positive cells. Dot plots (PI on *y* axis vs. AV on *x* axis) representative of five independent experiments are also shown. **(D, E)**. Bad and Bax expressions of FLS treated with rapamycin (200 nM for 4 h) and tofacitinib (1 uM for 20 h) analyzed by Quantitative Real-time PCR (qRT-PCR). Data are reported as differences between their Ct and the housekeeping gene GAPDH Ct (ΔCt). **(F)**. Western blot and densitometry analysis of citrullinated proteins levels in FLS from RA patients cultured in the presence of tofacitinib/rapamycin alone or in combination. Figure was chosen as representative of those obtained from five independent experiments on FLS from different RA patients. *p = 0.02.

First, we investigated whether the addition of the JAKs inhibitor tofacitinib in cell cultures of FLS obtained from RA patients could affect autophagy and apoptosis. Based on preliminary dose-response experiments performed on FLS isolated from OA patients (Supplementary Figure S1), cells were cultured in the presence of tofacitinib at a concentration of 1uM for 20 h. *In vitro* experiments were performed using a previous experimental setting consisting in the pre-incubation of FLS with rapamycin to induce autophagy and the subsequent addition of tofacitinib to the culture for 20 h (Vomero et al., 2019). Western blot analysis showed a reduction of LC3-II after treatment with tofacitinib (Figure 1A). As a confirmation of autophagy inhibition, an accumulation of p62, a selective substrate for autophagy (Ichimura et al., 2008) was also noticed (Figure 1A). The pre-treatment with rapamycin activated autophagy and the subsequent incubation with tofacitinib decreased levels of autophagy marker LC3-II as shown by Western blot analysis (Figure 1A). These results were also confirmed by flow cytometry (Figure 1B). Regarding apoptosis, tofacitinib did not significantly change the percentage of AV-positive FLS (Figure 1C). Also, the expression of the apoptosis-related genes Bad and Bax was not influenced by treatment with tofacitinib (Figures 1D,E). We further analyzed the presence of citrullinated proteins following autophagic stimulus in FLS from patients with RA and with OA. The results revealed a significant increase of citrullinated proteins in cells from RA patients compared with those from OA patients (data not shown). Nevertheless, western blot analysis of RA cells treated with tofacitinib did not show a significant modulation of citrullination compared to untreated cells (Figure 1F).

**FIGURE 2:**
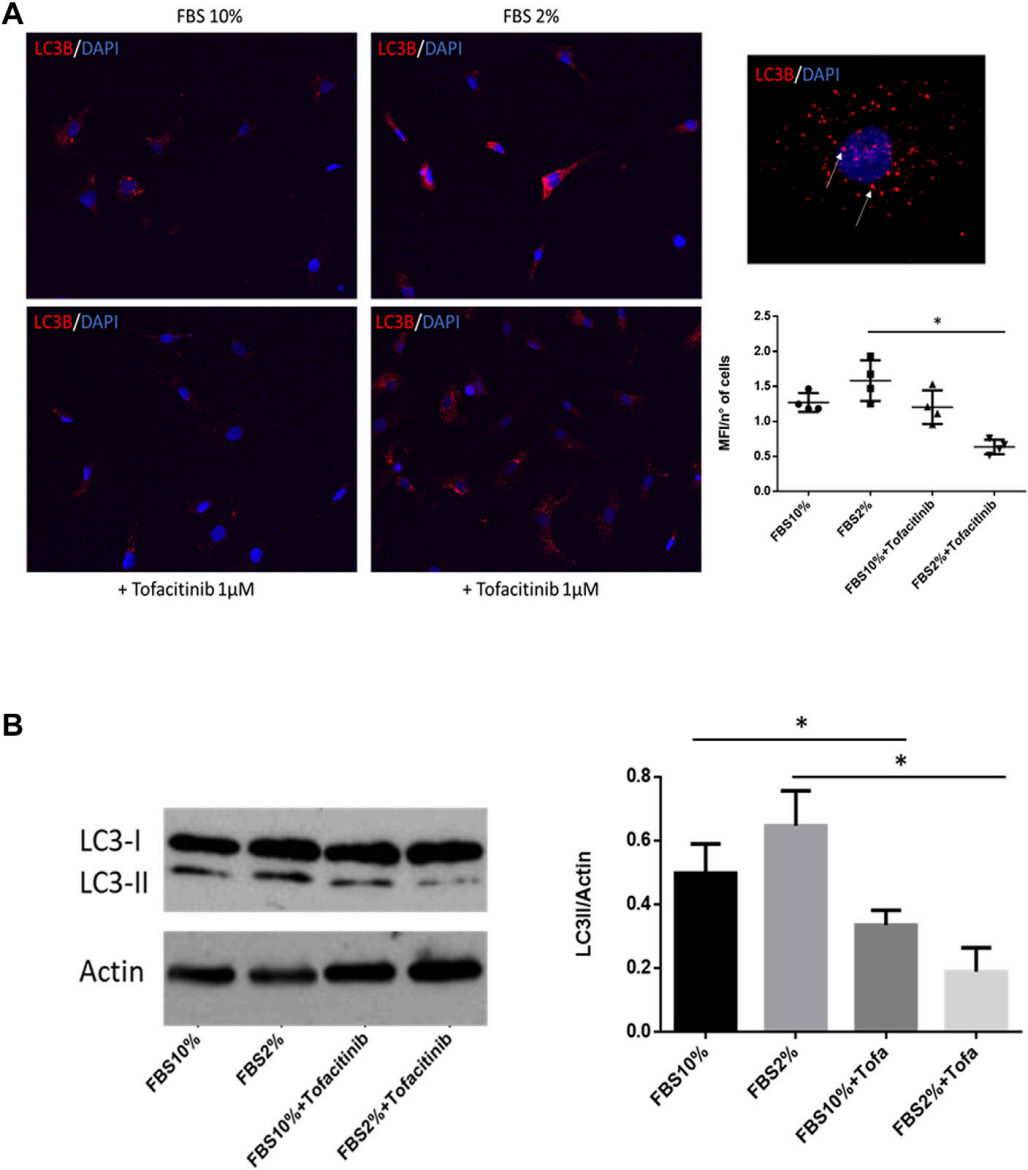
Effect of tofacitinib on autophagy in FLS cultured in serum deprivation condition. **(A)** Immunocytofluorescence analysis of autophagy in FLS treated with tofacitinib alone and in starvation (FBS 2%). Autophagic LC3B + vesicles are stained in red in RA FLS treated with tofacitinib alone and in serum deprivation condition for 20 h (2% FBS). Cells were counterstained with DAPI dye to reveal DNA (blue staining). Autophagy is expressed as MFI normalized on the number of cells. The quantification was performed on four different areas for each condition. Images are representative of five independent experiments and higher magnification (×40) picture is also showed. *p < 0.05. **(B)** Western blot analysis of autophagy levels in FLS cultured in the presence of tofacitinib. Starvation was used to induce autophagy (FBS 2% for 20 h), and tofacitinib was used as a treatment for 20 h (1 µM). Autophagy was quantified by densitometric analysis on LC3-II and then normalized on β-Actin. Data are referred to five independent experiments in cells from different RA patients. *p < 0.05.

Since RA autophagy is involved in FLS survival during stress stimuli, we also evaluated the effect of tofacitinib on autophagy induced by starvation by culturing FLS in serum-deprived medium (2% FBS). As shown in immunocytofluorescence staining, the percentage of autophagic vacuoles was increased in the cells cultured in starvation, and this autophagy induction was prevented by adding tofacitinib (Figure 2A). Furthermore, tofacitinib reduced levels of LC3-II in western blot in FLS cultured in serum deprivation condition (Figure 2B).

## Discussion

This study provides the first evidence that the JAK inhibitor tofacitinib could decrease autophagy of FLS from RA patients. This experimental evidence underlines how autophagy activation is crucial for the survival of inflammatory synoviocytes in RA.

Tofacitinib is a first-generation JAK inhibitor blocking the downstream signal of cytokines whose receptors use JAK3/JAK1 and to a lesser extent JAK2 (Tanaka and Yamaoka, 2013). The precise effect of tofacitinib on different cells involved in RA pathogenesis is largely unknown. In particular, given that the survival of aggressive synoviocytes is considered a key element in the RA pathogenesis, the modulation of FLS autophagy by tofacitinib could explain its therapeutic effects. Recent evidence suggested a role of autophagy in apoptosis decrease of RA synoviocytes and peripheral immune cells (Xu et al., 2013; Vomero et al., 2019). To our knowledge, this is the first study reporting the ability of tofacitinib to downregulate autophagy in synoviocytes from patients affected by RA.

Increased autophagy levels in synovial fibroblasts from RA patients were reported and a possible protective role of autophagy against apoptosis was suggested (Shin et al., 2010). The interaction between JAK/STAT and PI3K/AKT/mTOR axis was involved on the prevention of apoptosis. In fact, it has been demonstrated that the cytokines-induced JAK/STAT pathway contribute to the transcriptional regulation of Bcl-2 family members (Steelman et al., 2008).

Nevertheless, since we have not shown a modulation of apoptosis of RA-FLS, it could be plausible that other signals are needed to activate apoptosis *in vitro*. This result is partially in agreement with others that showed different proliferation and apoptosis balance of synovial cells in RA patients treated with tofacitinib (Maeshima et al., 2012; Ando et al., 2016).

Citrullination, a chemical conversion of arginine in citrulline by the action of peptidylarginine deiminase enzymes, has a crucial role in RA pathogenesis. In FLS from RA patients, the citrullinated protein levels increased after treatment with the autophagy inducer rapamycin highlighting a possible role of autophagy in the break of self-tolerance (Sorice et al., 2016). In this study, we confirmed this result; however, modulation of the autophagic process by tofacitinib *in vitro* did not show a significant change in citrullinated proteins. It remains to be clarified whether other post-translational protein modifications could be affected by tofacitinib.

In conclusion, the results of this study elucidated a new mechanism of action of tofacitinib related to autophagy/apoptosis modulation leading to a better understanding of the possible role of autophagy as a therapeutic target in RA.

## Data Availability

The raw data supporting the conclusion of this article will be made available by the authors, without undue reservation.

## Supplementary Material

Supplementary Figure S1 | Dose-response experiments performed on FLS isolated from OA patients. Western blot image of LC3-II protein levels in OA FLS treated with tofacitinib at concentration of 0.1, 0.5 and 1 uM for 20 h. Where indicated, cells were cultured in the presence of DMSO used to resuspend tofacitinib. Densitometry analysis of results obtained from five independent experiments is also shown * p=0.01.
