# Supplementary figures and images for: Tofacitinib Decreases Autophagy of Fibroblast-Like Synoviocytes From Rheumatoid Arthritis Patients
Source: Front Pharmacol. 2022 Mar 3;13:852802. doi: 10.3389/fphar.2022.852802 (PMC8928732; doi:10.3389/fphar.2022.852802)

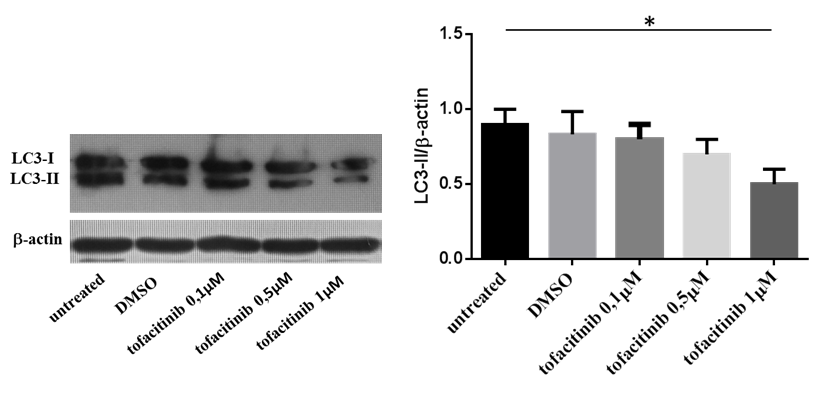

Supplement: Supplementary file 1 [file Image1.tif]
